# Prioritizing genes for systematic variant effect mapping

**DOI:** 10.1093/bioinformatics/btaa1008

**Published:** 2020-12-10

**Authors:** Da Kuang, Rebecca Truty, Jochen Weile, Britt Johnson, Keith Nykamp, Carlos Araya, Robert L Nussbaum, Frederick P Roth

**Affiliations:** btaa1008-aff1 Donnelly Centre, University of Toronto, Toronto, ON M5S 3E1, Canada; btaa1008-aff2 Department of Molecular Genetics, University of Toronto, Toronto, ON M5S 1A8, Canada; btaa1008-aff3 Lunenfeld-Tanenbaum Research Institute, Sinai Health System, Toronto, ON M5G 1X5, Canada; btaa1008-aff4 Department of Computer Science, University of Toronto, Toronto, ON M5T 3A1, Canada; btaa1008-aff5 Invitae Corporation, San Francisco, CA 94103, USA

## Abstract

**Motivation:**

When rare missense variants are clinically interpreted as to their pathogenicity, most are classified as variants of uncertain significance (VUS). Although functional assays can provide strong evidence for variant classification, such results are generally unavailable. Multiplexed assays of variant effect can generate experimental ‘variant effect maps’ that score nearly all possible missense variants in selected protein targets for their impact on protein function. However, these efforts have not always prioritized proteins for which variant effect maps would have the greatest impact on clinical variant interpretation.

**Results:**

Here, we mined databases of clinically interpreted variants and applied three strategies, each building on the previous, to prioritize genes for systematic functional testing of missense variation. The strategies ranked genes (i) by the number of unique missense VUS that had been reported to ClinVar; (ii) by movability- and reappearance-weighted impact scores, to give extra weight to reappearing, movable VUS and (iii) by difficulty-adjusted impact scores, to account for the more resource-intensive nature of generating variant effect maps for longer genes. Our results could be used to guide systematic functional testing of missense variation toward greater impact on clinical variant interpretation.

**Availability and implementation:**

Source code available at: https://github.com/rothlab/mave-gene-prioritization

**Supplementary information:**

Supplementary data are available at *Bioinformatics* online.

## 1 Introduction

Clinical genetic testing is frequently performed for carrier screening and for the detection and diagnosis of disease. The rapid decline in sequencing costs, coupled with increased demand for genetic testing and a shift in the standard of care toward larger gene panels, has resulted in rapid increases in the number of previously unseen variants (Blazer *et al.*, 2015).

Clinical variant interpretation is required to determine which variants are worthy of a physician’s concern. In ClinVar, a widely used resource for reporting clinical variants, ∼40% of all variants reported in disease-implicated genes are missense variants (Landrum *et al.*, 2016). Unfortunately, more than half of these missense variants are classified as variants of uncertain significance (VUS) (Starita *et al.*, 2017; Weile and Roth, 2018). Although physicians and genetic counselors do flag VUS emerging from a diagnostic genetic test for future follow-up and reinterpretation, VUS results are generally not considered clinically actionable (Hoffman-Andrews, 2017). Therefore, evidence that can help reclassify VUS as ‘benign’, ‘likely benign’, ‘pathogenic’ or ‘likely pathogenic’ could substantially impact the value of genetic testing for patient care. Although well-established functional assays, such as complementation (Lee and Nurse, 1987; Osborn and Miller, 2007) or *in vitro* biochemical activity assays (Guidugli *et al.*, 2014; Millot *et al.*, 2012), are used as evidence for variant interpretation under current American College of Medical Genetics and Genomics/Association for Molecular Pathology (ACMG/AMP) guidelines (Richards *et al.*, 2015; *Brnich et al., 2018*; Brnich *et al.*, 2019), such assays are resource-intensive and have not generally been performed for rare clinical missense variants. Multiplexed assays of variant effects (MAVEs) are an emerging tool to provide systematic experimental testing of nearly all missense variants for selected protein targets (Starita *et al.*, 2017). Missense variant effect maps have, for some genes, been shown to outperform general computational predictors of pathogenic variation (previously trained on variants across many genes) (Sun *et al.*, 2020; Weile *et al.*, 2017). For example, a variant effect map for the human *CBS* gene was generated in yeast cells based on the ability of each variant to complement the loss of the yeast *CBS* ortholog *CYS4*. This MAVE study detected almost four times more pathogenic variants than a computational approach, at the same stringency (Sun *et al.*, 2020).

Because MAVE technology has emerged only recently (Fowler and Fields, 2014), full-length variant effect maps are available for fewer than 30 human proteins (Esposito *et al.*, 2019; Weile and Roth, 2018). Moreover, many of these maps cover proteins for which very few difficult-to-interpret missense VUS have been reported. As the output of MAVE studies rapidly increases (Weile and Roth, 2018), it would be helpful to have more guidance on the protein targets for which variant effect maps could have the greatest impact on clinical variant interpretation. Here, we implement three strategies, each building on the previous, to prioritize gene targets of missense variant effect mapping studies.

## 2 Materials and methods

### 2.1 Clinical variants

We used ClinVar (Landrum *et al.*, 2016) to assemble missense variants that had been interpreted in the context of clinical genetic testing (‘clinical testing’), as opposed to variants in the literature that were not clearly interpreted in the context of clinical testing (‘literature-only’), and had been assigned a clinical interpretation of ‘uncertain significance’ by all submitters. Variants with conflicting interpretations were removed.

Missense variants were also extracted from Invitae’s clinical variant database, as were the number of patients in which each variant was found, the clinical area of the patients’ tests, and the most recent interpretation of each variant. Because our repeated observation (i.e. reappearance) measure could potentially over-weight genes that had been subject to more intensive cascade screening of individuals related to the primary proband (i.e. extensive family member testing), we included only variants from probands who were not known to be related. Variants were interpreted using the Sherloc system, for which both methods and validation have been published (Nykamp *et al.*, 2017). Interpreted variants were stripped of all protected health information (i.e. de-identified) under an approved protocol from the Western Institutional Review Board (IRB #20161796). As previously described, the Sherloc system offers a series of detailed refinements to the ACMG/AMP variant classification criteria to capture exceptions and special cases. For all VUS in the database, we retrieved the semiquantitative scores that had been assigned given supporting evidence types. Briefly, each evidence criterion was awarded a preset number of points on the benign (1B-5B) and pathogenic (1P-5P) scales, and total benign and pathogenic points were calculated separately (see Nykamp *et al.*, 2017 for details on the Sherloc system). To be classified as ‘likely benign’ or ‘likely pathogenic’, a variant had to receive at least three benign points (i.e. 3B) or four pathogenic points (i.e. 4P), respectively. For instance, a variant that is absent from the Exome Aggregation Consortium (ExAC), a large-scale reference human genetic variation dataset (Lek *et al.*, 2016), would receive 1P, and another 3P if it were seen in four unrelated clinical case reports, so that it would receive a total of four pathogenic points and be classified as ‘likely pathogenic’, in the absence of other evidence.

### 2.2 Accounting for ‘movability’ of variants

Variants annotated as VUS were considered ‘movable’ if new functional evidence (i.e. evidence from functional assays) would be sufficient to change the variant interpretation to a non-VUS category. We computed the number of movable VUS under two scenarios: functional evidence was ‘strong’ and functional evidence was ‘weak’, conveying either two and a half points or one point, respectively, within the Sherloc system (Nykamp *et al.*, 2017). For VUS to be ‘moved’, a variants’s total (after considering new strong or weak functional evidence) would need to either reach four pathogenic points (4P) or three benign points (3B). For example, VUS previously receiving two pathogenic points (2P) and no benign points (0B) based on other scoring criteria, if found to significantly reduce fitness in a human cell line assay and if the interpreter considered this to be strong functional evidence worth 2.5P, would now have a total of 4.5P and be moved to ‘likely pathogenic’. Alternatively, variants that previously received no pathogenic or benign points (0P, 0B) could not reach 4P even with the same piece of strong functional evidence, and would not be considered ‘movable’.

This analysis accounted for pre-existing functional evidence (e.g. VUS for which strong functional evidence had already been considered in the classification would not be considered movable). Any VUS with substantial evidence for a benign classification (>3B) and substantial evidence for a pathogenic classification (>4P) was not considered movable, because new functional evidence is unlikely to resolve the conflict.

### 2.3 Giving extra weight to reappearing variants

To score the tendency of a gene to harbor clinical variants that are repeatedly observed, we divided (for each gene) the number of patients in which a variant had been observed by the number of unique variants observed for that gene. To reduce the impact of common variants on our analysis, we capped the number variant observations at seven (treating variants seen in more than seven patients as though they had been observed in only seven). Applying this cap, 95% of VUS were left unchanged (Supplementary Fig. S1a).

### 2.4 Modeling the reappearance-weighted fraction of movable variants

We used the Invitae clinical variant database to calculate two coefficients that were subsequently used to modify variant effect map impact estimates for each gene. The first coefficient, the fraction of all variants that were movable (i.e. movability fraction, or *M*), was calculated as:

(1) $$M\left(g\right)=\frac{C_{\mathrm{movable}}\left(g\right)}{C\left(g\right)},$$

where C(g) is the unique VUS count for gene g and $C_{\mathrm{movable}}(g)$ is the unique movable VUS count for gene g. To limit the effects of small sample size for genes with fewer observed variants, we used a more conservative estimation approach (i.e. regularized movability fraction, or $\hat{M}$) that returned values closer to the average M value ($M_{\mathrm{avg}}$) where less data were available. This was calculated using:

(2) $$\hat{M}\left(g\right)=\frac{C_{\mathrm{movable}}\left(g\right)+C_{\mathrm{pseudo}}\cdot M_{\mathrm{avg}}}{C(g)+C_{\mathrm{pseudo}}},$$

where C(g) is the unique VUS count for gene g and $C_{\mathrm{pseudo}}$ is a pseudocount (see next page for a description of pseudocount selection) added to allow processing genes for which no movable VUS were observed in the Invitae dataset.

The second coefficient we calculated gives extra weight for repeated observations (R) of a variant. This ‘reappearance coefficient’, which captures the ratio of patients to unique variants while limiting the extra weight provided by capping the number of occurrences, was calculated by:

(3) $$R\left(g\right)=\frac{P\left(g\right)}{C\left(g\right)},$$

where P(g) is the number of patients observed to have a VUS for gene g. To limit the effects of small sample size for genes for which fewer patients had been observed, we calculated a regularized R ($\hat{R}$) that returned values closer to the average R value ($R_{\mathrm{avg}}$) when less data were available:

(4) $$\hat{R}\left(g\right)=\frac{P\left(g\right)+C_{\mathrm{pseudo}}\cdot R_{\mathrm{avg}}}{C(g)+C_{\mathrm{pseudo}}},$$

where C(g) and $C_{\mathrm{pseudo}}$ are the unique VUS count for gene g and a pseudocount, respectively.

For each regularization step, we used $C_{\mathrm{pseudo}}=8$. Thus, e.g. a gene with only two observed VUS had an M or R value that was 80% [8 pseudocounts/(8 pseudocounts + 2 observed VUS) = 80%] driven by the average value across all genes, while M or R for a gene with more than 72 VUS was more than 90% [72 observed VUS/(8 pseudocounts + 72 observed VUS) = 90%] driven by VUS observed in that gene. A pseudocount enabled the model to process datasets (e.g. ClinVar) where movability fraction (M) and reappearance (R) are not available, as long as a dataset (e.g. Invitae) is present to provide the average movability and reappearance.

Our simplest measure of impact score ($S_{\mathrm{ClinVar}}$) was calculated simply as the unique VUS count:

(5) $$S_{\mathrm{ClinVar}}\left(g,C\right)=C\left(g\right),$$

where C(g) is the unique VUS count for gene g in ClinVar. We then used the above-described coefficients of movability and reappearance to calculate a movability- and reappearance-weighted impact score (MARWIS) ($S_{\mathrm{MARWIS}}$) for each gene with at least one VUS in the ClinVar database:

(6) $$S_{\mathrm{MARWIS}}\left(g,C\right)=\hat{M}\left(g\right)\cdot \hat{R}\left(g\right)\cdot C\left(g\right),$$

where C(g) is the unique VUS count for gene g. MARWIS was calculated either using the count of unique VUS from ClinVar ($C_{\mathrm{ClinVar}}$) or from Invitae ($C_{\mathrm{Invitae}}$), yielding $S_{\mathrm{MARWIS}}\;(g,C_{\mathrm{ClinVar}})$ and $S_{\mathrm{MARWIS}}\;(g,C_{\mathrm{Invitae}})$, respectively.

In calculating $S_{\mathrm{ClinVar}}$ and $S_{\mathrm{MARWIS}}$ scores, the goal was to estimate absolute impact on clinical variant interpretations that a variant effect map might provide, so we did not consider measures of difficulty of the project, e.g. by considering the length of each protein.

### 2.5 Difficulty-adjusted impact score (DAIS)

For each protein, a DAIS ($S_{\mathrm{DAIS}}$) was calculated to account for the increased difficulty of variant effect mapping for longer proteins:

(7) $$S_{\mathrm{DAIS}}\left(g,C\right)=\frac{S_{\mathrm{MARWIS}}\left(g,C\right)}{D_{L}\left(g\right)+D_{\mathrm{fixed}}},$$

where $D_{L}(g)$ is the protein length [i.e. number of amino acids, based on the canonical isoform according to the Ensembl database (Yates *et al.*, 2019)]. The denominator estimated the resources that would be required for variant effect mapping, as the sum of length-dependent ($D_{L}$) and -independent ($D_{\mathrm{fixed}}$) costs, with length-independent costs modeled as equivalent to the length-dependent costs of a 300 amino acid protein ($D_{\mathrm{fixed}}=300$). Length-independent costs were introduced to capture costs such as assay development and assay validation experiments, as well as length-independent aspects of downstream analysis.

### 2.6 Prioritization of genes

**Fig. 1.**
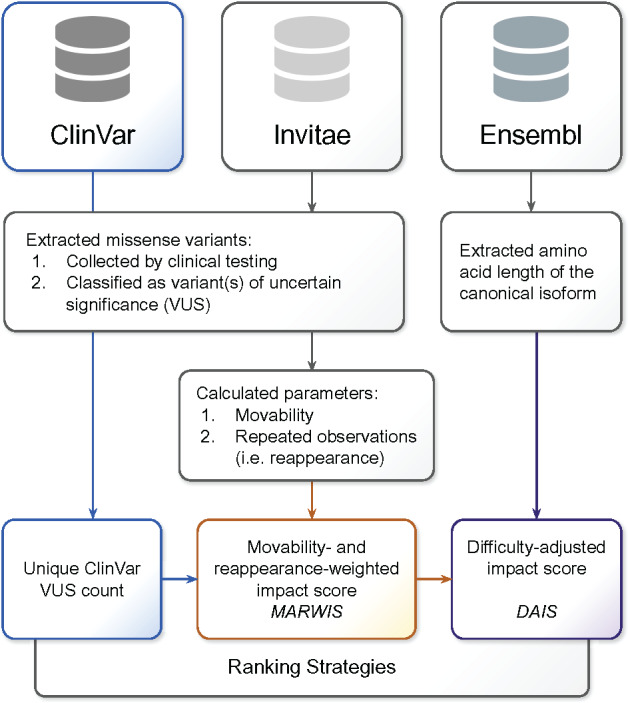
Three strategies for ranking genes according to the potential impact of variant effect map on clinical interpretation of VUS. Missense VUS collected through clinical testing were extracted from the ClinVar and Invitae databases. The first strategy ranked genes based on their unique VUS count. The second strategy ranked genes based on their MARWIS to give extra weight to reappearing, movable VUS. The third strategy ranked the genes by their DAIS, calculated to account for the costs associated with studying longer genes

Figure 1 shows the three strategies we used to prioritize genes according to how useful a variant effect map could be in classifying variants for those genes. The first strategy ranked the genes by the number of unique missense VUS that had been reported to ClinVar (‘VUS count’). The second strategy ranked genes by MARWIS to give extra weight to reappearing, movable VUS. The third strategy ranked genes by DAIS, which rescale the MARWIS to account for the more resource-intensive nature of generating variant effect maps for longer genes. Analysis scripts, datasets and raw results are available via GitHub (https://github.com/rothlab/mave-gene-prioritization).

## 3 Results

### 3.1 Application of prioritization strategies

The three strategies—using (i) ‘unique VUS count’, based on the number of unique VUS in ClinVar; (ii) MARWIS, the ClinVar VUS count modified by Invitae-derived movability and reappearance coefficients and (iii) DAIS, which adjusted the MARWIS with a rough estimate of the relative difficulty of producing a variant effect map—resulted in three unique lists of the 20 highest-priority genes (Table 1).

**Table 1.** Top 20 genes ranked by strategy

Rank	Unique ClinVar VUS	MARWIS	DAIS
1	*TTN*	***ATM***	*TP53*
2	***BRCA2***	***BRCA2***	*MYH7*
3	***ATM***	*MYH7*	*CHEK2*
4	***APC***	***NF1***	***MSH2***
5	***MSH6***	*TTN*	***MSH6***
6	***NF1***	***APC***	*VHL*
7	***BRCA1***	***POLE***	***ATM***
8	***POLE***	***MSH6***	*MLH1*
9	***MSH2***	***BRCA1***	***BRCA2***
10	***PALB2***	*RYR1*	***BRCA1***
11	*TSC2*	***MSH2***	***NF1***
12	***BRIP1***	*FBN1*	*MUTYH*
13	*RYR2*	***PALB2***	***PALB2***
14	*DICER1*	***BRIP1***	*STK11*
15	*PMS2*	*CHEK2*	*POLD1*
16	*PLEC*	*POLD1*	***POLE***
17	*RAD50*	*DICER1*	***BRIP1***
18	*BARD1*	*TP53*	*NBN*
19	*SYNE1*	*MLH1*	*PMS2*
20	*CDH1*	*TSC2*	***APC***

Genes in **bold** were common to all three top 20 lists.

For the ‘unique VUS count’ approach, we examined 221 538 unique missense VUS in 3646 genes from ClinVar (accessed on September 2, 2020). This approach to prioritization had the advantage of being simple and readily calculated from available ClinVar data. However, this approach had two limitations. First, it counted VUS even where new functional evidence would not lead to reclassification. Second, it gave no extra weight to VUS that appeared in multiple patients, even though reclassification of such variants would have greater clinical impact. Unfortunately, these issues could not be addressed using only the public ClinVar resource, which does not provide information about movability. Although ClinVar can contain multiple reports for the same variant, the number of such reports is a poor proxy for the rate of repeated observation, given that providers will typically only provide a new report to ClinVar upon observation in a new patient if their interpretation has changed.

To obtain information about movability and reappearance for each VUS, we mined data from the Invitae database, which encompassed 411 782 missense VUS observations (218 096 unique missense VUS in 1921 genes). Following ACMG/AMP guidelines, as implemented by the Sherloc system (Nykamp *et al.*, 2017) used by Invitae for interpretation, we established that in the Invitae database, only 9.6% of VUS were potentially movable on the basis of strong, new, functional evidence (3.7% to benign or likely benign, 6.9% to pathogenic or likely pathogenic and 1% to either category given the direction of functional evidence).

**Fig. 2.**
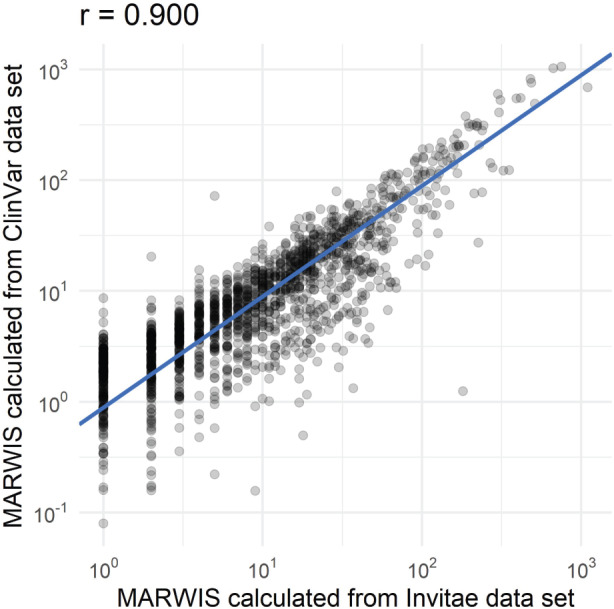
Correlation between MARWIS calculated from two datasets. The MARWIS calculated using unique missense VUS from ClinVar ($S_{\mathrm{MARWIS}}(C_{\mathrm{ClinVar}})$) correlated well (*r* = 0.900) with the MARWIS calculated using unique missense VUS from the Invitae dataset for 1921 genes ($S_{\mathrm{MARWIS}}(C_{\mathrm{Invitae}})$). The blue line shows the reduced major axis regression of the dataset

After calculating movability and reappearance coefficients for each gene (see Section 2), we combined the two coefficients with the VUS count from ClinVar to estimate an MARWIS for each of the 3646 genes with missense VUS in ClinVar. To limit the dependence of gene prioritization on the experience of any one clinical genetics provider, we only used ClinVar variants to derive the unique VUS count. However, results based on unique VUS counts from Invitae yielded similar MARWIS (Pearson correlation, *r* = 0.90) (Fig. 2). We applied reduced major axis regression, which is more appropriate than ordinary least-square regression when a bivariate relationship is symmetrical (Smith, 2009).

**Fig. 3.**
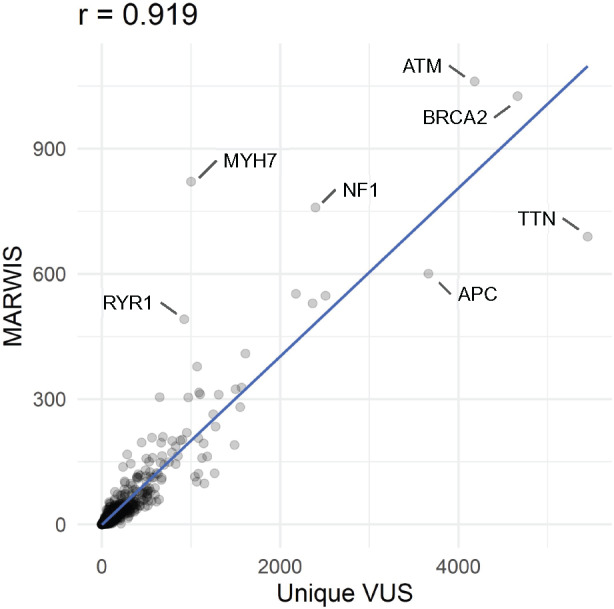
Correlation between unique VUS count and MARWIS. The unique VUS count correlated well (*r* = 0.919) with the MARWIS. Some genes, including *RYR1*, *NF1*, *MYH7*, *ATM* and *BRCA2*, exhibited more frequently reappearing and movable VUS than the group average, whereas other genes (e.g., *TTN* and *APC*) showed fewer. The blue line shows the linear regression of the data

Ranking ClinVar genes by unique VUS count and by MARWIS yielded substantial agreement (Pearson correlation, *r* = 0.92) (Fig. 3), but with many noteworthy exceptions. For most genes that were extreme outliers, the MARWIS were higher than those for other genes with similar unique VUS counts, indicating that consideration of variant reappearance and movability can substantially alter prioritization. For example, as shown in Figure 3, the *MYH7* MARWIS was nearly five times the score projected from the *MYH7* unique VUS count by the linear regression model (y = 0.2017c -1.1323, where c is the unique missense VUS count from ClinVar). *MYH7* variants are associated with familial hypertrophic cardiomyopathy (CMH1 [MIM: 192600]) and dilated cardiomyopathy (CMD1S [MIM: 613426]) (Fiorillo *et al.*, 2016). Although hundreds of causal variants (e.g. LRG_384p1: p.Arg403Gln) have been reported (Geisterfer-Lowrance *et al.*, 1990), 30–40% of CMH1 (Arad *et al.*, 2005; Watkins *et al.*, 2008) and 65–80% of CMD1S (Daehmlow *et al.*, 2002) cases cannot be explained by known pathogenic variants. Correspondingly, about 70% of all missense variants in the *MYH7* gene in ClinVar have been annotated as VUS. Because 49.7% of *MYH7* variants are moveable, as opposed to 9.6% of VUS overall, evidence of variant functionality might be expected to offer a greater-than-average benefit for clinical interpretation of *MYH7* variants.

Although the MARWIS approach allows ranking of genes for which a variant effect map would provide the greatest impact on clinical variant interpretation, it does not account for the idea that producing a variant effect map is more resource-intensive for a longer protein than for a shorter one. The top 20 MARWIS-ranked genes encoded proteins with an average length of 3674 amino acids (based only on the annotated canonical isoform), as compared with an average length of 842 amino acids over all proteins scored.

Therefore, we developed the DAIS, which divides the MARWIS by a gene-specific estimate of relative difficulty. This estimate was necessarily crude, as determining the best functional assay (e.g. *in vitro* test versus complementation in human cells) for each disease-associated protein and its relative cost is not currently feasible. A detailed analysis for limitation of this estimation and ways to further prioritize ranked genes are explored in Section 4.

### 3.2 Comparison of rankings

Examining the ‘top 20’ genes from each of the three lists, we found 10 genes common to all lists: *BRCA2*, *ATM*, *APC*, *MSH6*, *NF1*, *BRCA1*, *POLE*, *MSH2*, *PALB2* and *BRIP1* (Table 1). (‘top 100’ lists are also provided in Supplementary Table S1.) For other genes, ranks differed substantially between the lists. For example, the *TP53* gene was ranked 48th by unique VUS count, 18th by MARWIS and 1st by DAIS. *TP53* was the top DAIS-ranked gene, though the DAIS still likely underestimated the impact of somatic variation (see Section 4). In keeping with its top rank by DAIS, multiple MAVE studies (Bhagavatula *et al.*, 2017; Giacomelli *et al.*, 2018; Kotler *et al.*, 2018) have been conducted to better understand*TP53* variation.

Ranking genes by DAIS provided an additional aspect of gene prioritization that would otherwise have been ignored. For example, the *VHL* gene was ranked 6th by DAIS despite being 36th by MARWIS and 137th by unique VUS count. Variation in *VHL* can cause familial erythrocytosis (ECYT2 [MIM: 263400]), von Hippel–Lindau syndrome (VHL [MIM: 193300]) and clear cell renal carcinoma (CCRC [MIM: 144700]) (Gossage *et al.*, 2015). Its importance in genetic disease already suggested that it should be a high-priority target for proactive functionality testing, but the fact that it is a relatively short protein (178 amino acids) suggests that it should be further prioritized. The ‘top 40’ list for this preferred strategy is provided in Table 2, annotated by previously conducted systematic variant mapping studies, number of unique missense variants and VUS in ClinVar, typical mode of inheritance, whether or not pathogenic variants tend to be gain-of-function or loss-of-function, and the clinical categories to which each gene belongs.

**Table 2.** Top 40 genes ranked by DAIS

Rank	Gene	Unique missense variants in ClinVar (# and % that are missense)	Mode of inheritance and molecular mechanism	Clinical categories
1	*TP53* ^a^	1091 (649, 59%)	Autosomal dominant—Li-Fraumeni syndrome (LoF)	Exome, hereditary cancer, preventive
2	*MYH7*	1432 (1001, 70%)	Autosomal dominant—dilated cardiomyopathy, hypertrophic cardiomyopathy, left ventricular non-compaction Autosomal recessive—distal myopathy	Cardiology, exome, preventive
3	*CHEK2*	1328 (1090, 82%)	Autosomal dominant—CHEK2-related cancer risk (LoF)	Hereditary cancer, preventive
4	*MSH2^b^*	2118 (1611, 76%)	Autosomal dominant—Lynch syndrome (LoF) Autosomal recessive—constitutional mismatch repair deficiency syndrome (LoF)	Exome, hereditary cancer, preventive
5	*MSH6*	3115 (2509, 81%)	Autosomal dominant—Lynch syndrome (LoF) Autosomal recessive—constitutional mismatch repair deficiency syndrome (LoF)	Exome, hereditary cancer, preventive
6	*VHL*	478 (285, 60%)	Autosomal dominant—von Hippel–Lindau syndrome (LoF) Autosomal recessive—familial erythrocytosis (LoF)	Exome, hereditary cancer, preventive
7	*ATM*	5000 (4181, 84%)	Autosomal dominant—ATM-related cancer risk (LoF) Autosomal recessive—ataxia-telangiectasia (LoF)	Carrier, hereditary cancer, preventive
8	*MLH1*	1341 (970, 72%)	Autosomal dominant—Lynch syndrome (LoF)	Exome, hereditary cancer, preventive
9	*BRCA2*	7433 (4663, 63%)	Autosomal dominant—hereditary breast and ovarian cancer syndrome (LoF) Autosomal recessive—Fanconi anemia (LoF)	Exome, hereditary cancer, preventive
10	*BRCA1* ^c^	4333 (2364, 55%)	Autosomal dominant—hereditary breast and ovarian cancer syndrome (LoF)	Exome, hereditary cancer, preventive
11	*NF1*	3412 (2396, 70%)	Autosomal dominant—neurofibromatosis, type 1 (LoF)	Hereditary cancer, pediatric genetics, preventive
12	*MUTYH*	790 (665, 84%)	Autosomal recessive—*MUTYH*-associated polyposis (LoF)	Exome, hereditary cancer, preventive
13	*PALB2*	2039 (1568, 77%)	Autosomal dominant—PALB2-related cancer risk (LoF) Autosomal recessive—Fanconi anemia (LoF)	Hereditary cancer, preventive
14	*STK11*	704 (568, 81%)	Autosomal dominant—Peutz-Jeghers syndrome (LoF)	Exome, hereditary cancer, preventive
15	*POLD1*	1150 (1102, 96%)	Autosomal dominant—MDPL syndrome (LoF), POLD1-related cancer risk (LoF)	Hereditary cancer, preventive
16	*POLE*	2273 (2176, 96%)	Autosomal dominant—POLE-related cancer risk (LoF) Autosomal recessive—FILS syndrome (LoF)	Hereditary cancer, preventive
17	*BRIP1*	1760 (1500, 85%)	Autosomal dominant—BRIP1-related cancer risk (LoF) Autosomal recessive—Fanconi anemia (LoF)	Hereditary cancer, preventive
18	*NBN*	1137 (952, 84%)	Acute lymphoblastic leukemia (LoF) Autosomal recessive—Nijmegen breakage syndrome (LoF)	Carrier, hereditary cancer, preventive
19	*PMS2*	1566 (1275, 81%)	Autosomal dominant—Lynch syndrome (LoF) Autosomal recessive—constitutional mismatch repair deficiency syndrome (LoF)	Exome, hereditary cancer, preventive
20	*APC*	4381 (3663, 84%)	Autosomal dominant—familial adenomatous polyposis (LoF)	Exome, hereditary cancer, preventive
21	*RAD51C*	611 (539, 88%)	Autosomal recessive—Fanconi anemia (LoF)	Hereditary cancer, preventive
22	*BMPR1A*	622 (530, 85%)	Autosomal dominant—juvenile polyposis syndrome (LoF)	Exome, hereditary cancer, preventive
23	*RAD51D*	466 (397, 85%)	Autosomal dominant—RAD50-related cancer risk (LoF) Autosomal recessive—Nijmegen breakage syndrome-like disorder (LoF)	Hereditary cancer
24	*CDH1*	1329 (1142, 86%)	Autosomal dominant—hereditary diffuse gastric cancer (LoF)	Hereditary cancer, preventive
25	*RAD50*	1409 (1250, 89%)	Autosomal dominant—RAD50-related cancer risk (LoF) Autosomal recessive—Nijmegen breakage syndrome-like disorder (LoF)	Hereditary cancer
26	*PTEN* ^d^	652 (394, 77%)	Autosomal dominant—Cowden syndrome (LoF)	Exome, hereditary cancer, preventive
27	*CDKN2A*	401 (337, 84%)	Autosomal dominant—melanoma (LoF)	Hereditary cancer, preventive
28	*LMNA*	551 (324, 59%)	Autosomal dominant—Hutchinson-Gilford progeria syndrome, congenital muscular dystrophy, dilated cardiomyopathy (LoF), Emery-Dreifuss muscular dystrophy (LoF), Limb-Girdle muscular dystrophy (LoF), lipodystrophy Autosomal recessive—Charcot-Marie-Tooth disease, type 2 (LoF), Emery-Dreifuss muscular dystrophy	Exome, neurology, preventive
29	*BARD1*	1354 (1182, 87%)	Autosomal dominant—breast cancer (LoF)	Hereditary cancer, preventive
30	*AXIN2*	908 (852, 94%)	Autosomal dominant—AXIN2-related carcinoma (LoF)	Hereditary cancer, preventive
31	*DICER1*	1486 (1310, 88%)	Autosomal dominant—Pleuropulmonary blastoma (LoF)	Hereditary cancer, preventive
32	*TSC2*	2366 (1552, 66%)	Autosomal dominant—tuberous sclerosis-2 (LoF)	Exome, pediatric genetics, preventive
33	*MYBPC3*	1112 (685, 62%)	Autosomal dominant—dilated cardiomyopathy, hypertrophic cardiomyopathy, left ventricular non-compaction	Cardiology, exome, preventive
34	*TNNT2*	228 (147, 64%)	Autosomal dominant—familial hypertrophic cardiomyopathy (Lof) Autosomal dominant—dilated cardiomyopathy (LoF)	Cardiology, exome, preventive
35	*SDHB*	362 (255, 70%)	Autosomal dominant—gastrointestinal stromal tumor syndrome (LoF), paraganglioma–pheochromocytoma syndromes (LoF), SDH-related renal cell carcinoma (LoF) Autosomal recessive—mitochondrial complex II deficiency (LoF)	Exome, hereditary cancer, preventive
36	*MEN1*	634 (409, 65%)	Autosomal dominant—multiple endocrine neoplasia (LoF)	Exome, hereditary cancer, preventive
37	*FH*	492 (319, 75%)	Autosomal recessive—Fumarase deficiency (LoF)	Hereditary cancer, preventive
38	*MSH3*	887 (787, 89%)	Autosomal recessive—familial adenomatous polyposis (LoF)	Hereditary cancer, preventive
39	*FBN1*	2475 (1069, 43%)	Autosomal dominant—Marfan syndrome (LoF)	Cardiology, exome, preventive
40	*LDLR*	982 (237, 24%)	Autosomal dominant—familial hypercholesterolemia (LoF)	Cardiology, exome, preventive

Diseases were categorized as loss-of-function (LoF) or gain-of-function (GoF) depending on the underlying molecular mechanism. Small superscript letters indicate systematic variant effect studies for each gene.

a *TP53*: Bhagavatula *et al.* (2017), Giacomelli *et al.* (2018) and Kotler *et al.* (2018).

b *MSH2*: Jia *et al.* (2020) on bioRxiv.

c *BRCA1*: Findlay *et al.* (2018), Starita *et al.* (2015) and Starita *et al.* (2018).

d *PTEN*: Matreyek *et al.* (2018) and Mighell *et al.* (2018).

### 3.3 Sensitivity of results to parameter choices

To assess whether our results were sensitive to the parameters of our analysis, pseudocounts ranging from 1 to 10 were explored. High correlation between rankings generated with different pseudocount choices suggested that our results were not highly sensitive to pseudocount choice (Supplementary Fig. S2, lowest observed *r* = 0.999). Next, we varied the choice of cap on the number of repeated observations from 1 to 10. High correlation between ranks suggested that our results were also not highly sensitive to this choice (Supplementary Fig. S1b, lowest observed *r* = 0.96). Finally, length-independent costs of a protein ranging from 0 amino acids (i.e. no length-independent cost) to 500 amino acids (assuming length-independent costs are equivalent to the length-dependent costs for a protein of length 500) were explored. Our rankings were also not highly sensitive to the choice of length-independent cost (Supplementary Fig. S3, lowest observed *r* = 0.94).

## 4 Discussion

Previous studies have provided metrics to rank human genes based on how likely they are to tolerate functional genetic variation (Lek *et al.*, 2016; Petrovski *et al.*, 2013). Despite being useful for inferring gene essentiality, such measures do not predict the burden of clinical missense variants annotated as VUS. Indeed, there are many examples of absolutely essential genes that are not associated with genetic disease (e.g. because defects yield embryonic lethality).

Here, we examined three strategies for ranking genes according to the potential for MAVEs to assist clinical variant interpretation, and further provided a list of prioritized genes using each strategy. We recommended the most nuanced DAIS strategy because it takes into account protein length as at least one element of difficulty in variant effect mapping.

Some of the prioritized genes we identified had already been subjected to systematic functional testing. For example, a recent MAVE study on functionally critical domains of the *BRCA1* gene identified more than 400 non-functional missense variants that might improve clinical interpretation of *BRCA1* variants (Findlay *et al.*, 2018). High throughput assays performed on the *TP53* gene (Bhagavatula *et al.*, 2017; Giacomelli *et al.*, 2018; Kotler *et al.*, 2018) revealed novel loss-of-function and gain-of-function missense variants that might facilitate interpreting *TP53* clinical variants. However, variant effect mapping studies are currently available for only 4 of the top 40 DAIS-ranked genes (Table 2). To our knowledge, no variant interpretation currently reported in ClinVar has yet made use of MAVE evidence.

As the MAVE community continues publishing high-quality MAVE studies, it is potentially beneficial to re-evaluate the effectiveness of improving clinical variant interpretation with MAVE when more MAVE data become available.

We note that our ranking schemes, which sought to maximize impact on clinical variant interpretation, should not be confused with rankings by overall clinical impact of variant interpretation. Such a ranking would also need to consider clinical actionability. A more complex accounting of the clinical impact gained by improved clinical interpretation would be a valuable future direction. Although this would be an enormous undertaking that falls outside the scope of this work, we expect that the scoring schemes described here could contribute to such an effort.

Another limitation was that we did not separately account for the effect of somatic variants when estimating impact scores. Although some of the variants reported in ClinVar have been observed somatically, most are germline variants (Landrum *et al.*, 2018). While *TP53* is one of the most frequently somatically mutated genes in human cancers (Olivier *et al.*, 2010), with missense variants reported in more than 25 000 cancer samples in the Catalogue of Somatic Mutations in Cancer (Tate *et al.*, 2019), somatic variants represent only 74 (11%) of the 649 missense VUS for *TP53* in ClinVar. Thus, our prioritization largely ignored the impact that variant effect maps could have on interpreting somatic variants. Prioritizing genes based on a more inclusive combination of somatic and germline variation, as well as other variant types (e.g. in-frame deletions or insertions, intronic or promoter variation) is an avenue for future investigation.

We did not consider reasons to study the functional impact of human variation beyond that of improving clinical variant interpretation. For example, MAVE studies can provide clues about functional protein domains (e.g. by revealing potential protein interaction interfaces) (Weile *et al.*, 2017).

Our study calculated movability in terms of what fraction of VUS could potentially be reclassified with the appearance of strong new functional evidence. Of course, the number and identity of movable variants will ultimately depend on the quality of the new functional evidence and on the variant interpreter’s judgment (following ACMG/AMP guidelines) of the strength of evidence it provides. As a result, the impact of a variant effect map on clinical interpretation should ultimately be assessed for each individual gene and map. As such analysis emerges, we imagine that any trends could be considered in *a priori* prioritization of other genes (e.g. to upweight priorities for well-conserved metabolic enzymes if it were found that maps for these genes tended to provide stronger evidence).

It would also be of interest to determine the genes and variants for which systematic gathering of other types of evidence (e.g. co-segregation of phenotypes within families), either alone or together with functional evidence, could affect variant classification. This type of analysis could also consider synergism between functional evidence and other evidence types. For example, systematic analysis of co-segregation could not only enable reclassification for some VUS, but also place others within range of reclassification based on new functional evidence, thereby increasing the impact of a variant effect map.

Although current ACMG/AMP guidelines make no recommendations as to whether variant effect maps provide strong or weak evidence for benign or pathogenic classification, published studies have demonstrated the value of variant effect maps in identifying pathogenic variants (Sun *et al.*, 2020; Weile *et al.*, 2017). However, although this study defined a VUS to be movable to pathogenic/likely pathogenic or to benign/likely benign given strong functional evidence, no similar studies have investigated the value of these maps in identifying benign variants with confidence. Thus, the determination of movability should be revisited as a better understanding of the evidentiary performance of MAVEs emerges.

Our prioritization strategies implicitly considered reclassification from VUS to pathogenic/likely pathogenic to have the same clinical value as reclassification from VUS to benign/likely benign. However, future ranking schemes might place more clinical value on reclassification toward pathogenicity. Our model also did not account for the fact that VUS in known cancer driver genes might have more clinical impact than a VUS in a gene where disease association has not been firmly established. In theory, a less disease-relevant gene with many VUS could be ranked higher than a known disease-causing gene with very few VUS. However, genes for which a disease association is not clear are less likely to be included in clinical genetic tests; as a result, these genes should have fewer VUS identified and hence lower prioritization by our ranking strategies.

Although we recommend DAIS as it balances benefits against costs, the estimation of difficulty used in DAIS was necessarily crude, as no systematic measure is available to determine the best functional assay for each disease-associated protein and its relative cost. Therefore, after identifying a ‘short list’ of genes, users may wish to reweight MARWIS based on their own estimates of relative difficulty. There are other ways to refine the relative priorities of genes that were top-ranked by one of our scores. For instance, one might prefer essential genes in more tractable cell lines given the relative ease of assay development. When multiple alternative functional assays are available, users may wish to separately adjust not only for the difficulty of each alternative assay, but also by basing weight on expected fidelity or evidentiary value of that assay. However, because such criteria tend to vary between users, they were not included as general adjustment parameters in this study.

Because the quality of an MAVE study depends on the validity of the functional assays it uses, a quality assurance step is required to benchmark the robustness of functional assays by evaluating a list of variants known to be pathogenic or likely pathogenic and variants known to be benign or likely benign. As a result, in Supplementary Table S1, we provide (i) the number of unique pathogenic, likely pathogenic, likely benign and benign variants in ClinVar and (ii) the number of unique VUS, the number of occurrence of VUS, the number of movable VUS and the number of occurrence of movable VUS in the Invitae database for the top 100 genes ranked by DAIS. A threshold might be applied to further prioritize genes with enough variants with clear clinical classification. To facilitate functional assay selection, we developed MaveQuest, an online resource for planning experimental tests of human variant effects (Kuang *et al.*, 2020). For example, for *CHEK2*, which was ranked third by DAIS, MaveQuest suggests multiple potential functional assays. One possibility is a CRISPR knockout assay supported by a negative viability phenotype observed in a systematic CRISPR knockout screen (Wang *et al.*, 2015). Another is a trans-species complementation assay based on the ability of human *CHEK2* to complement the loss of the yeast ortholog *RAD53* (Roeb *et al.*, 2012).

Calculation of MARWIS was only possible given coefficients derived from data on movability and repeated observations of VUS, such as those provided by Invitae. Although VUS counts were based on the more inclusive ClinVar dataset, genes for which Invitae offers tests had greater opportunity for increased (or decreased) prioritization based on movability and reappearance. We note that the composition of variants in ClinVar necessarily inherits biases from Invitae and major submitters to ClinVar. These biases could be viewed as a feature rather than a limitation, in that they should collectively tend to favor genes for which genetic assays are of greatest clinical interest. However, different clinical genetic services may differ in terms of their focus on particular genes and disease areas (e.g. somatic variation within tumor genomes). The modeling process we describe can be tuned to blend coefficients from multiple sources. Therefore, other organizations could enhance this prioritization process through similar sharing of de-identified clinical variation data collected under approved protocols.

In conclusion, our study explored three ways to prioritize genes according to the clinical impact of systematically testing missense variant functions. Through use of the DAIS strategy in particular, we offer a list of genes prioritized by potential benefit in clinical variant interpretation, as weighted by a measure of difficulty in producing the maps. As more VUS are identified in annotated disease genes, and as more genes are implicated in disease, the priority list of genes will evolve. Therefore, the prioritization exercise reported here should be periodically revisited.

## Supplementary Material

btaa1008_Supplementary_Data
