# Supplementary material for: Prioritizing genes for systematic variant effect mapping
Source: Bioinformatics. 2020 Dec 10;36(22-23):5448–55. doi: 10.1093/bioinformatics/btaa1008 (PMC8016487; doi:10.1093/bioinformatics/btaa1008)
Supplement: btaa1008_Supplementary_Data [file btaa1008_supplementary_data.zip › Supplementary Figures.pdf]

Supplementary Figures

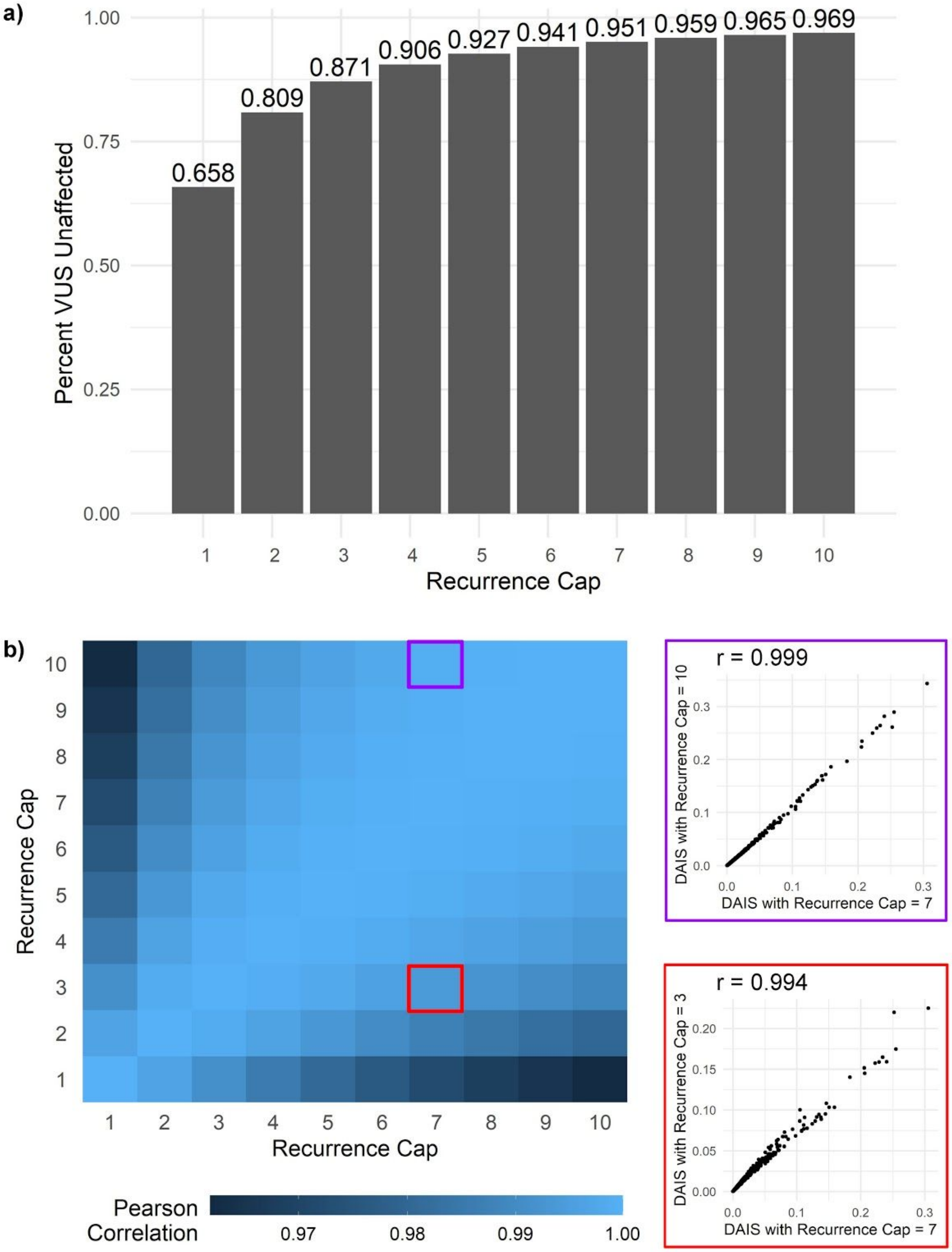

Fig. S1. Sensitivity of DAIS-based ranking strategy to reappearance caps. **a)** We considered VUS that had been seen in more than seven patients to have been observed in only seven patients (i.e. a reappearance cap of seven). This was selected because 95% of VUS were unaffected given such a cap. **b)** We compared DAIS from datasets with different reappearance caps (from 1 -10) and calculated Pearson Correlation (PCC) to evaluate if different reappearance caps impact the ranking strategy.

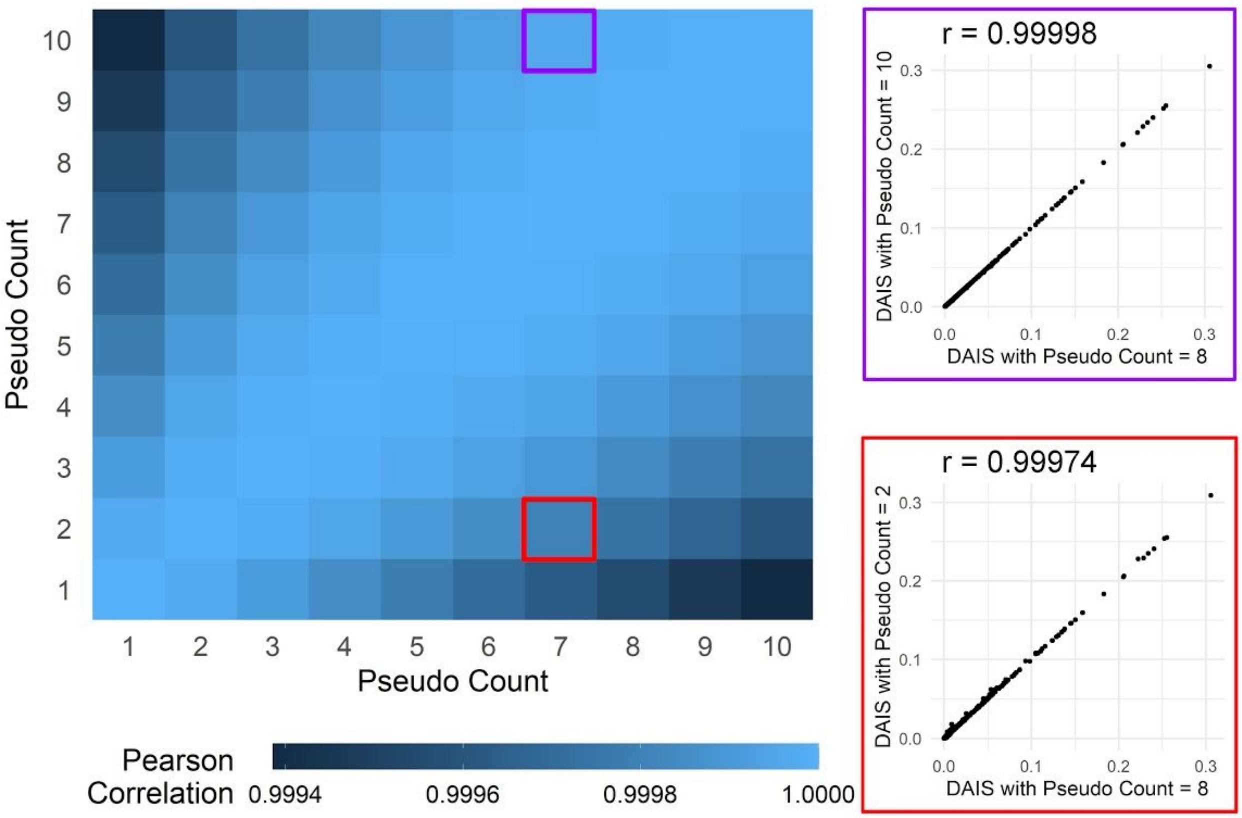

Fig. S2. Sensitivity of DAIS-based ranking strategy to pseudo counts. We considered a range of pseudo counts from 1 to 10 when calculating the regularized movability fraction and reappearance (see Materials and Methods section) and evaluated if different pseudo counts impact the ranking strategy.

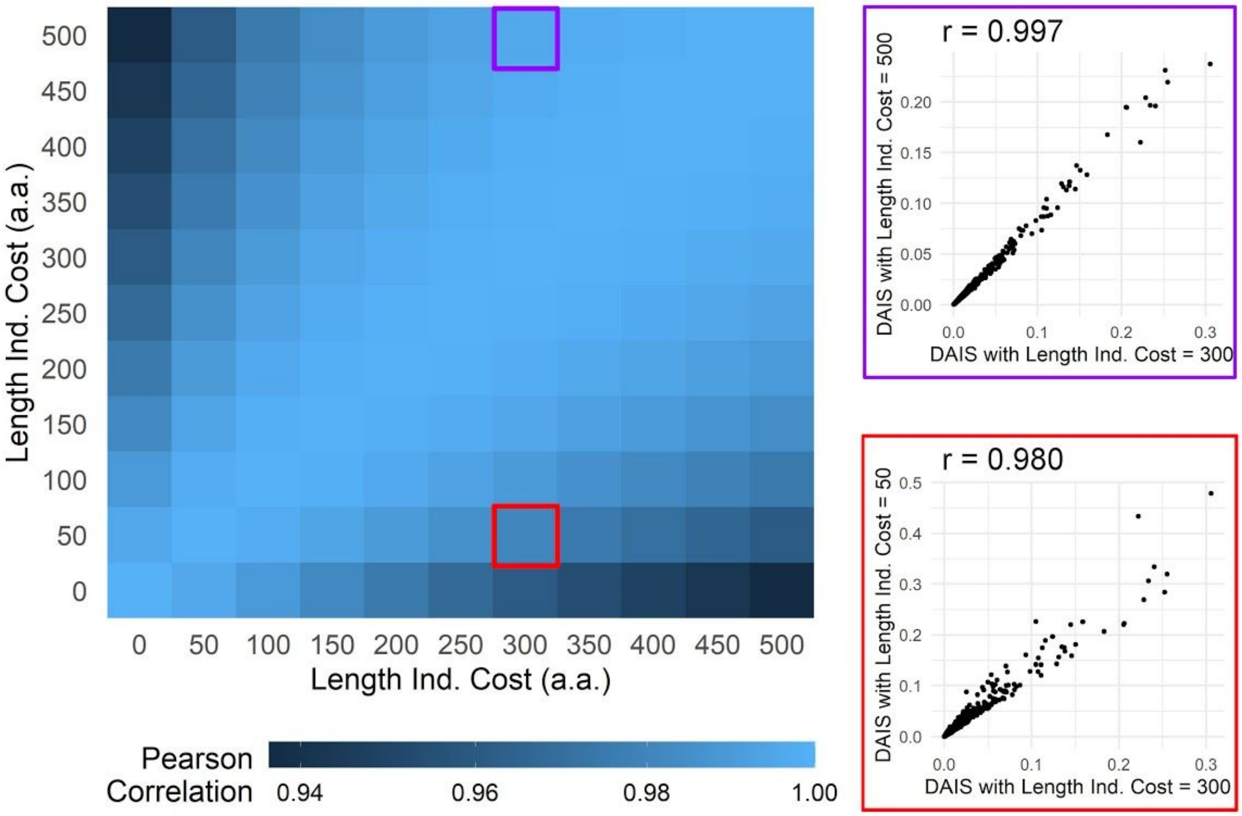

Fig. S3. Sensitivity of DAIS-based ranking strategy to length-independent cost. We considered a range of length-independent costs modeled as equivalent to the length-dependent costs of a protein from 0 amino acids (i.e. no length-independent cost) to 500 amino acids a protein with from 0 to 500 and evaluated if different length-independent costs impact the ranking strategy.
